# Quality of life in benign colorectal disease—a review of the assessment with the Gastrointestinal Quality of Life Index (GIQLI)

**DOI:** 10.1007/s00384-023-04473-y

**Published:** 2023-06-20

**Authors:** Karl-Hermann Fuchs, Frauke Musial, Laura Retzbach, Alexander Hann, Alexander Meining

**Affiliations:** 1 Laboratory for Interventional and Experimental Endoscopy (InExEn), Gastroenterology, University of Würzburg, Auvera-Haus, Grombühlstr.12, 97080 Würzburg, Germany; 2 National Research Center in Complementary and Alternative Medicine (NAFKAM), Department of Community Medicine, UiT, The Arctic University of Norway, Tromsø, Norway

**Keywords:** Benign colorectal disease, Quality of life, Gastrointestinal Quality of Life Index, GIQLI, Diverticulitis, Constipation, Fecal incontinence

## Abstract

**Background and purpose:**

The Gastrointestinal Quality of Life Index (GIQLI) is an instrument for the assessment of quality of life (QOL) in diseases of the upper and lower GI tract, which is validated in several languages around the world. The purpose of this literature review is the assessment of the GIQLI in patients with benign colorectal diseases. Reports on GIQLI data are collected from several institutions, countries, and different cultures which allows for comparisons, which are lacking in literature.

**Methods:**

The GIQL Index uses 36 items around 5 dimensions (gastrointestinal symptoms (19 items), emotional dimension (5 items), physical dimension (7 items), social dimension (4 items), and therapeutic influences (1 item). The literature search was performed on the GIQLI and colorectal disease, using reports in PubMed. Data are presented descriptively as GIQL Index points as well as a reduction from 100% maximum possible index points (max 144 index points = highest quality of life).

**Results:**

The GIQLI was found in 122 reports concerning benign colorectal diseases, of which 27 were finally selected for detailed analysis. From these 27 studies, information on 5664 patients (4046 female versus 1178 male) was recorded and summarized. The median age was 52 years (range 29–74.7). The median GIQLI of all studies concerning benign colorectal disease was 88 index points (range 56.2–113). Benign colorectal disease causes a severe reduction in QOL for patients down to 61% of the maximum.

**Conclusions:**

Benign colorectal diseases cause substantial reductions in the patient’s QOL, well documented by GIQLI, which allows a comparison QOL with other published cohorts.

## Introduction

The clinical appearance of colorectal diseases shows a large variety depending on the underlying cause [1–4]. Different entities can be varying in their influences on the patient’s discomfort and their restriction in daily life. In addition, the individual symptom load and coping abilities of given patients add to the variable reductions in quality of life (QOL) of individuals [1, 5].

Furthermore, QOL is an important factor characterizing the patient’s status, which can be quantitatively assessed and which can be used in medicine as an integrating parameter, which summarizes all multifactorial influences on the patient’s condition at a certain time point or time segment, when it is applied and evaluated [6–9]. Therefore, an increasing interest has emerged in QOL instruments as an outcome parameter of patients, using the integral ability of describing the condition of a given individual.

One of these frequently used instruments of QOL assessments is the Gastrointestinal Quality of Life Index (GIQLI) [5, 10]. The GIQLI is an established instrument for clinical evaluation and research in gastrointestinal (GI) diseases and was established and first validated in German and subsequently in English language [5, 10]. Currently, the GIQLI has been validated for several other languages [11–16]. The latter allows for a broad application of this instrument in research in different countries and cultures, which allows for interesting comparisons [10–16].

GIQLI assessment has been broadly used in the surgical literature to determine and compare the pre- and postoperative status of the patient’s QOL [1–4, 10–23]. One further advantage of the GIQLI is that it is easy to answer and no supervision of the patients while filling in the forms is required [5, 10, 17, 23]. Besides a global mean, the GIQLI provides 5 different dimensions (gastrointestinal symptoms; the emotional, physical, and social factors; and a therapeutic component), allowing for a more detailed analysis of the patient’s QOL [5, 10, 17, 23]. Thus, the GIQLI is also suitable to monitor the patient’s social support and network throughout the course of the disease and its treatment. In addition, the psycho-emotional component of the patient’s condition can be monitored next to the spectrum of symptoms.

Furthermore, GIQLI data have also been generated in healthy non-patient cohorts, which allows for comparison of patient data with these control cohorts [5, 10, 17]. The latter fact has shown to be of advantage, since these data provide an effective tool for comparing the patient’s QOL status with a normal level as well as before and after any therapy [1, 5, 10–23].

However, GIQLI data are lacking, comparing the QOL status among different investigated cohorts of the same disease entity as well as comparing different patient cohorts with related disease entities such as the group of benign colorectal diseases.

Therefore, the purpose of this study is a literature review on cohorts of patients with benign colorectal disease, which have been evaluated prior to therapy by the application of the GIQLI. We were interested in the analysis of patient cohorts from different institutions and countries with respect to disease severity and other possible factors influencing QOL assessed with the GIQLI. In addition, these data can be compared to GIQLI data from a healthy non-patient population [5, 10, 17].

## Methods

This analysis was part of a larger project, investigating the published data on the application of GIQLI between 1995 and 2022 in several gastrointestinal diseases [23]. For this current study, a literature search was performed for reports on the application of GIQLI in pubmed.gov, using the search terms [GIQLI] and [GIQLI and colorectal disease]. Primary inclusion criteria were reports on GIQLI-measurements in patients with benign colorectal diseases prior to any invasive therapy. It must be emphasized that we strictly followed the original methodology of the GIQLI analysis as published and validated [5, 10, 17]. QOL was assessed by the GIQL index, which uses 36 items around 5 dimensions (gastrointestinal symptoms (19 items), emotional dimension (5 items), physical dimension (7 items), social dimension (4 items), and therapeutic influences (1 item)) [5, 10]. The possible maximum values for GIQLI indicating a high QOL were 76 index points for gastrointestinal symptoms, 20 index points for the emotional dimension, 28 index points for the physical dimension, 16 points for the social dimension, and 4 index points evaluating the influence of therapeutic components. In total, a person can reach a maximum of 144 index points (Table 1). The selected articles were screened for the correct application of the GIQLI and its dimensions. Publications which provided the correct analysis and presentation of GIQLI were further selected for the assessment in this study.

**Table 1:** GIQLI by Eypasch et al. (1995) in healthy volunteers of validation population from [5, 10, 17] and display of percentage of “normal” GIQLI from maximum index points

	*n*	Sex: female/male	Mean age	GI-symp	emot	phys	soc	ther	GIQLI mean
Volunteers	168	76/92	42	62	18.5	23.5	14.8	3.8	122.6
Maximum index-points of GIQLI				76	20	28	16	4	144
Percentage of maximum (%)				81	92	84	91	95	85

**Fig. 1:**
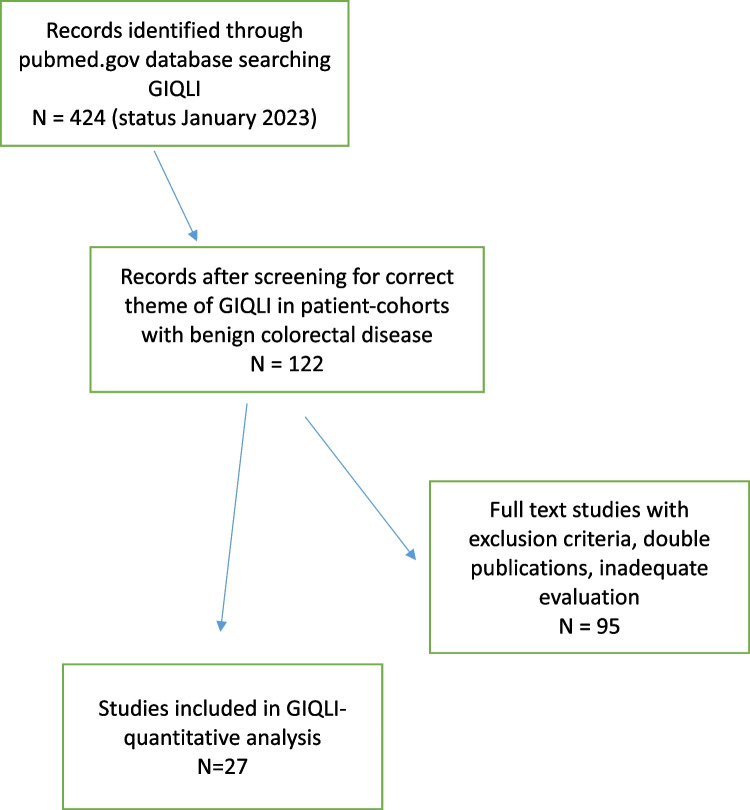
The selection process following the PRISMA guidelines

The selection process followed the PRISMA guidelines as shown in Fig. 1 [24]. The abstracts were screened for data on GIQLI in patient cohorts with benign colorectal disease. Articles with double publications of cohorts, inadequate use of the validated methodology of the GIQLI, or any other deviation from the correct use and application of GIQLI were excluded. Quite frequently, published studies showed an evaluation, which did not follow the strict assessment rules of the GIQLI. In addition to the GIQLI data, available data on patient characteristics such as age, gender, and specific parameters of the disease entity were documented.

In order to facilitate comparison of the level of GIQLI points between different study cohorts, absolute GIQLI points as well as the percentage of these index points of the maximally possible GIQLI points were determined.

For the comparison of the study results with a healthy, non-patient control group, the original publication of Eypasch et al. was used [5, 10, 17]. The percentage of the GIQLI points of the results from the normal population and the patient cohorts was then compared with regard to the maximum GIQLI level as well as for each dimension. These results are presented descriptively.

## Results

### Control data from normal population

The total GIQLI for the 168 normal participants (122.6 points from a maximum of 144 index points) was obtained from Eypasch et al. [5, 10] (Table 1). The results for the different dimensions were as follows: gastrointestinal symptoms (GIsym) 62; emotional dimension (emot) 18.5; physical dimension (phys) 23; social dimension (soc) 14.8; and therapeutic influence (ther) 3.8. By calculating the percentage from the possible maximum index points, it can be seen that also a normal supposedly “healthy “ population is not likely to achieve total GIQLI points of 100% (maximum 144), since the data from Eypasch et al., popularized by Granderath et al., from a healthy, non-patient cohort show “only” 85–95% from the maximally possible points [5, 10, 17].

### GIQLI in patients with benign colorectal disease-cohorts

In total, 424 publications are registered at pubmed.gov (status January 2023) on GIQLI search. After the PRISMA process, 27 publications were considered for further analysis (Fig. 1) [1–4, 25–47]. Others were excluded for double publication, mostly inadequate or non-complete evaluation of the GIQLI according to the original methodology as published and validated. In addition, many studies focused only on postoperative results after surgical therapy and did not include any preoperative data regarding the disease.

From the total of 27 studies selected, information on 5664 patients (4046 female versus 1178 male) was recorded and summarized in Table 2 [1–4, 25–47]. Unfortunately, the vast majority of the publications did not provide the necessary detailed calculations for the different dimensions of the GIQLI. Therefore, only the overall GIQLI points could be analyzed and demonstrated in this current study. The median value of the cohorts regarding age was 52 years with a cohort range of 29–74.7 years. The median GIQLI of all studies concerning benign colorectal disease was 88 index points (range 56.2–113). With maximum of GIQLI points being 144, the presence of benign colorectal disease causes a severe reduction in QOL for patients down to 61% of the maximum in the overall cohort. Data from only 10 articles allowed for an evaluation of the different dimensions among all cohorts. Only one statement could be made regarding the emotional dimension, which shows in various disease cohorts a median of 11 index points and thus a reduction to 55% of the possible maximum of 20 index points.

**Table 2:** Overview of selected publications on GIQLI in benign colorectal disease

Author/year	Diagnosis of cohort	*n*	Sex f/m	Mean age years	Mean GIQLI	Range GIQLI
Sailer et al. (1998) [1]	Benign colorectal	325	143/182	49	113	93–120
Damon et al. (2004) [2]	Constip + fecal incon	157	138/19	54	89	86–92
Coffin et al. (2004) [25]	IBS	858	591/267	-	88	84–93
Casellas et al. (2005) [26]	Celiac disease	54	40/14	35	86.4	
Maartense et al. (2006) [27]	M.Crohn	60	34/26	30	83	
Leger et al. (2008) [28]	Fecal incontinence	92	0/92		81	
Seneviratne et al. (2009) [29]	Fistula	21		32.4	97	
Forgione et al. (2009) [30]	Diverticulitis	46	20/26	58.3	99.5	
Pasternak et al. (2012) [31]	Diverticulitis	120	76/54	59	95	88–107
Adusumilli et al. (2013) [32]	Fecal incontinence	142		60	93	
Damon et al. (2013) [33]	Fecal incontinence	102	99/3	59	71.3	
Gosselink et al. (2013) [34]	Constipation	42	40/2	55	83	
Maggiori et al. (2013) [35]	Constip/prolaps	33	29/4	64	77	
Duchalais et al. (2015) [36]	Constipation	21	17/4	47	69	
Mishra et al. (2016) [37]	Fecal incontinence	78		57	78	
Yang et al. (2018) [38]	Constipation	30		74.7	63.2	
Roman et al. (2019) [39]	Endometriosis	55	55/0	29	91	
Brochard et al. (2019) [40]	Constip + fecal incon	1870	1662/208	58.7	86.7	
Zhong et al. (2019) [41]	Constipation	49		41	82	
Ouizeman et al. (2020) [3]	Constip + fecal incon	422	345/77	59	90	
Yang et al. (2020) [42]	Constip + prolaps	130		50	101	
Santos et al. (2021) [43]	Diverticulitis	85	26/59	59	102	
Yang et al. (2021) [44]	Constipation	45	31/14	46	85	
Shahzad et al. (2021) [4]	Colorectal disease	199	84/115	42	102	
Mehedintu et al. (2021) [45]	Endometriosis	488	488/0	33	76.6	
Reh et al. (2022) [46]	Endometriosis	97	97/0		90.7	
Lin et al. (2022) [47]	IBS	43	31/12	35.2	56.2	

The focus on subgroups such as patients with constipation is demonstrated in Table 3 [1–3, 34–36, 38, 40–42, 44]. Eleven publications reported on this entity, providing the results of 1840 patients with constipation. The gender background shows 343 females and 62 males are involved. The median age of the cohort results is 51 years with a range of cohort means of 41–74.7 years. The median GIQLI of the cohorts is 85 index points (range 63.2–101), which represents 59% of the possible maximum index points.

**Table 3:** Overview of selected publications on GIQLI in patients with constipation

Author/year	*n*	Sex f/m	Mean age (years)	Mean GIQLI
Sailer et al. (1998) [1]	14			94
Damon et al. (2004) [2]	78	69/9	51	92.3
Maggiori et al. (2013) [35]	33	29/4	64	77
Gosselink et al. (2013) [34]	42	40/2	55	83
Duchalais et al. (2015) [36]	21	17/4	47	69
Yang et al. (2018) [38]	30		74.7	63.2
Brochard et al. (2019) [40]	1212			86
Zhong et al. (2019) [41]	49		41	82
Ouizeman et al. (2020) [3]	186	157/29	55	91
Yang et al. (2020) [42]	130		50	101
Yang et al. (2021) [44]	45	31/14	46	85
			Median	Median
Overall	1840	343/62	51	85
Range			41–74.7	63.2–101

A second subgroup, separately demonstrated in Table 4, are patients with fecal incontinence [1–3, 7, 8, 32, 33, 37, 40]. Eight publications focused on this entity covering 1525 patients. There were 366 females and 153 males. The median age of the cohorts was 59 years (range 57–60). The median GIQLI was 83.5 (range 71.3–93), which represents only 58% of the possible maximum index points and thus a remarkable reduction in QOL by this disease entity and devastating functional defect.

**Table 4:** Overview of selected publications on GIQLI in patients with fecal incontinence

Author/year	*n*	Sex f/m	Mean age (years)	Mean GIQLI
Sailer et al. (1998) [1]	35			93
Damon et al. (2004) [2]	79	69/10	57	86.8
Leger et al. (2008) [28]	92	0/92		81
Damon et al. (2013) [33]	102	99/3	59	71.3
Adusumilli et al. (2013) [32]	142		60	93
Mishra et al. (2016) [37]	78		57	78
Brochard et al. (2019) [40]	761			82
Ouizeman et al. (2020) [3]	236	198/48	60	85
			Median	Median
Cohort summary	1525	366/153	59	83.5
				Range 71.3–93

As a third subgroup the diagnosis of chronic diverticulitis was selected, which is a frequent diagnosis in GI disease; however, only 3 publications provided adequate data on GIQLI [30, 31, 43]. In total, data on 251 patients were published (female 122; male 139). The median age of the cohorts was 59 years. The cohorts median GIQLI was 99.5 (range 95–102), which represents 69% of the possible maximum index points.

The lowest level of GIQLI points were reported from several disease entities such as 71.3 (49.5% of the maximum) in a cohort of fecal incontinence, 76.6 (53.2% of the maximum) in a cohort of patients with endometriosis, 56.2 (39% of the maximum) in a cohort of patients with irritable bowel syndrome (IBS), and 63 (43.8% of the maximum) in a cohort of patients with constipation [33, 45, 47]. It can be stated as a summary of these results that patients with benign colorectal diseases may suffer from a major reduction in quality of life during the course of the disease, which requires optimal care to improve their situation.

## Discussion

The GIQLI is based on an instrument, which investigates not only symptoms in patients with gastrointestinal disease but is also able to differentiate between several dimensions such as emotional or psychologic components, the physical status of a patient, and the social relationships of a person, who may be quite dependent on such abilities, besides the influence of present GI symptoms [5, 10, 17, 23]. The majority of authors, using the GIQLI in patient cohorts with benign colorectal disease, have missed this rather unique opportunity to further move in the depth of the patient restriction by the disease by not providing this detailed information. As a result, the authors could only use the overall GIQLI points for the current analyses and interpretations. One could speculate that severe symptoms of fecal incontinence may have a large impact on the patient’s QOL, furthermore even a larger reduction in the dimension of emotional handling of such a devastating condition.

Overall, QOL is substantially reduced in patients with benign colorectal disease. The latter occurs in all entities, and this shows the potential of chronic colorectal disorders to deteriorate the patient’s life as time goes by [1]. This implicates the need for therapeutic decision making and often psychologic support [1, 2, 40, 48]. An accurate diagnostic assessment is needed, followed by an interdisciplinary exchange of information and an optimal selection of patients for surgical therapy, if this opens an option for improvement [1, 2, 30, 49–51]. Several reports demonstrate improvements in QOL by surgical therapy of chronic diverticulitis, fecal incontinence, and constipation [30, 49, 50].

The review shows that GIQLI can be used as an integral parameter reflecting the realistic patient’s condition—determined by herself/himself—and the involved restrictions, rather independent from specific disease parameters.

In addition, quality of life of a person is determined by many factors or dimensions, which may in some persons correlate with the severity of symptoms; in others, it may be multifactorial determined also by social and/or emotional influences as well [1–3, 27, 28, 30, 34, 49]. Therefore, the comparison of different studies on patients with benign colorectal disease, performed in different cultural environments from different countries, can be of substantial scientific and academic value. The integral values of index points, which have been generated in a standardized fashion, allow for comparison with both, other patient cohorts and published values from healthy, non-patient control cohorts [1, 2, 5, 10, 30, 34]. Comparing different clinical populations with different GIQLI points can provide an overview over the total span of symptom severity for the clinician, providing an additional tool for clinical decision-making [28, 30, 34, 49]. While a normal QOL level in patients with colorectal disease should encourage the managing therapists to be cautious in favoring a decision for interventional and surgical therapy, a substantial reduction in quality of life should motivate any physician to further investigate the reasons for such a deleterious QOL.

Using the level of QOL reduction for comparison with different disease entities may reveal some interesting aspects. Looking at patients with colorectal cancer shows the substantial level of reduction in QOL in some benign colorectal disorders such as fecal incontinence and constipation, since in the latter the GIQLI level remains between 80 and 90 index points, while in the published cancer series, the level is around 90 index points [1, 6, 18, 20, 33, 36, 38]. Very few disease entities show a comparable reduction in GIQLI of these just mentioned severe functional disabling entities, if one looks at gastroesophageal reflux disease, esophageal motility disorders, or gastric cancer [19–23].

The application of a validated instrument to assess QOL in patients with a given disease allows for a comparison of these patient’s QOL with a level of normal controls in order to make a judgment about the amount of QOL reduction this patient may suffer. Alternative instruments to assess QOL are available in literature [6, 8, 18, 49]. These complex clinical situations underline the necessity to investigate QOL in patients using a validated and widely used instrument such as the GIQLI. Using these validated instruments allows for the comparison of own data with other reports and especially with normal control data in literature. Systematic investigation and evaluation of QOL with a standardized instrument will improve daily clinical evaluations and more investigations in patient’s outcome.

## Conclusions

Benign colorectal diseases cause substantial reductions in the patient’s quality of life which can be well documented by the GIQLI. This instrument has been established in clinical medicine in the past 25 years especially among surgeons as a dependable tool to objectively assess the patient’s condition in various dimensions. This does provide a means to asses own patients with different diseases and in addition enables to compare QOL with those of other published cohorts; thus, it may provide a means to improve clinical practice.

## Data Availability

All data generated or analyzed during this study are included in this article. The data are published in literature as shown in the reference list. Further inquiries can be directed to the corresponding author.
